# Patient-reported outcome measure completion in lumbar spine surgery: Rates and determinants in a multicenter registry

**DOI:** 10.1016/j.bas.2026.106286

**Published:** 2026-08-18

**Authors:** Julia Mocking, Judith M.P. van Grafhorst, Christian Herrmann, Emin Aghayev, Carmen L.A. Vleggeert-Lankamp

**Affiliations:** a Department of Neurosurgery, Leiden University Medical Center, Leiden, the Netherlands; b EUROSPINE, the Spine Society of Europe, Uster, Switzerland; c Research Centre, Lindenhofgruppe AG, Bern, Switzerland; d Department of Neurosurgery, Spaarne Gasthuis, Haarlem/Hoofddorp, the Netherlands

**Keywords:** Patient-reported outcome measures, Response rate, Spine surgery, Registry data, Outcome assessment

## Abstract

**Introduction:**

Patient-reported outcome measures (PROMs) are increasingly used to aid clinical decision-making, evaluate treatment outcomes, and support quality improvement in spine care. However, to ensure valid and representative outcomes, sufficient completion rates are crucial.

**Research question:**

The objective of this study is to assess PROM completion rates in routine lumbar spine surgery care and to identify patient- and hospital-level factors associated with completion.

**Materials and methods:**

Data were obtained from patients undergoing low-complex lumbar spine surgery for degeneration, with and without instrumentation, recorded in the Spine Tango Registry. PROM completion was assessed as completion of any, baseline, or follow-up PROM, and paired baseline and follow-up PROMs. Mixed-effects logistic regression models were used to evaluate associations between PROM completion and patient characteristics (e.g. age, diagnosis, surgical procedure) and to describe hospital- and country-level variability. Additionally, an exploratory survey among participating hospitals assessed PROM collection strategies.

**Results:**

A total of 1810 patients from 5 countries were included. Overall, 67.3% of patients completed at least one PROM, 57.9% completed a baseline PROM, 53.3% completed a follow-up PROM and 44.4% completed paired baseline and follow-up PROMs. Completion rates declined with increasing follow-up duration. Age, diagnosis, and type of surgery were significantly associated with PROM completion. Substantial variability in PROM completion was observed between hospitals.

**Discussion and conclusion:**

PROM completion in routine lumbar spine surgery care remains suboptimal, particularly for long-term follow-up and paired PROMs. The large variability between hospitals indicates that hospital-level interventions are crucial to improve PROM completeness and ensure reliable outcome assessment.

## Highlights

• At least one PROM is completed by 67.3% of patients.
• Completion rates are highest at baseline and decline with follow-up duration.
• Age, diagnosis, and type of surgery are associated with PROM completion.
• PROM completion rates vary substantially across hospitals.

## 1 Introduction

The evaluation of healthcare outcomes has progressively shifted beyond clinical and radiological measures alone to include the patient perspective. While traditional assessments such as physical examinations and radiological imaging quantify anatomical or physiological changes, they do not necessarily capture the patients' experiences, including pain, functional limitations, and quality of life (Kluzek et al., 2022; Beyer et al., 2023; Khan et al., 2023). Patient-reported outcome measures (PROMs) address this gap by providing standardized and validated assessments of patients’ self-reported health (Churruca et al., 2021). This transition towards patient-centered outcome assessment is also evident in spine care, where PROMs are increasingly used for outcome evaluation (Khan et al., 2023; Guzman et al., 2016; Algattas et al., 2017).

By systematically capturing the patients’ perspective, PROMs provide valuable information across multiple levels of care (Calvert et al., 2019; Bonsel et al., 2024). At the individual level, they can support shared decision-making, enable monitoring of symptoms over time, and aid risk stratification (Calvert et al., 2019; Bonsel et al., 2024). At the same time, PROMs are used at the population level to evaluate the effectiveness of interventions, benchmark performance between healthcare providers, inform quality improvement initiatives, and assess cost-effectiveness (Churruca et al., 2021; Calvert et al., 2019; Bonsel et al., 2024). Consequently, PROMs are nowadays widely used in clinical research, routine outcome monitoring, and clinical registries (Calvert et al., 2019).

However, despite their growing use, the value of PROMs depends on the completion rates. Low completion not only hinders their applicability in individual patient care, but also results in non-response bias in research if patients who complete the questionnaires differ systematically from those who do not return the questionnaires. This threatens the representativeness of study populations and might result in biased estimates of treatment effectiveness or misleading comparisons between hospitals (Zini and Banfi, 2021). Recognizing these challenges, recent guidance on the use of patient-reported outcomes emphasizes the importance of carefully designing PROM collection procedures to maximize completion while minimizing participant burden, thereby supporting representative and interpretable PROM data (Cruz Rivera et al., 2022).

Achieving adequate PROM completion in routine care requires active engagement from both patients, who must allocate time and effort to complete the questionnaires, and hospitals, which are responsible for selecting PROMs, integrating PROMs into clinical workflows, distributing questionnaires, sending reminders, and training clinical staff regarding the use and interpretation of PROMs (Calvert et al., 2019; Glenwright et al., 2023). As a result, completion reflects both individual patient-level and systemic hospital-level influences. While limited single-center studies on PROM completion in spine surgery patients have been performed, these studies have reported highly variable completion rates and differing conclusions regarding associations between patient-level characteristics and PROM completion (Johnson et al., 2024; Ifearulundu et al., 2022; Vilkki et al., 2025). Moreover, due to their single-center nature, these studies could not assess variability in completion rates between hospitals. Although national spine registries have reported PROM completion rates (Swespine), to our knowledge, no multicenter studies have investigated the variation in PROM completion rates across hospitals and the association between completion and patient characteristics. As a result, the extent to which patient- and hospital-level factors influence PROM completion remains largely unknown.

Therefore, this study aimed to evaluate PROM completion in routine spine care using data from an international, multicenter spine registry. Specifically, we aimed to quantify completion rates in patients treated in hospitals that had implemented PROM collection systems, evaluate whether PROM completion is associated with patient characteristics, and assess the variation in completion rates between hospitals. Together, these findings may improve understanding of PROM completion in routine spine care and inform future strategies to optimize completion rates.

## 2 Methods

### 2.1 Study design and setting

This study is a retrospective multicenter observational cohort study using data from the EUROSPINE Spine Tango Registry, combined with a prospectively administered questionnaire completed by hospitals included in this study. The Spine Tango Registry is an international spine registry capturing data on diagnosis, treatment, and patient-reported outcomes from hospitals in Europe and beyond. The registry is hosted and operated by NEC Software Solutions UK. For the present study, registry data collected from patients treated in 2023 or 2024 were used.

### 2.2 Study population

Patients were included in this study if they met the following criteria: (1) had a diagnosis of lumbosacral radicular syndrome (LRS), lumbar spinal stenosis (LSS), or both, determined by their treating physician; (2) underwent decompression with or without fusion surgery; (3) received surgery after the date that the treating hospital initiated electronic PROM (ePROM) collection, which was defined as the first date on which an ePROM was sent by the hospital. This time point was used as a marker of formal PROM implementation within routine spine care at a hospital.

### 2.3 Outcomes

Baseline characteristics, including age, gender, diagnosis, number of affected segments, whether fusion surgery was performed, history of previous spine surgery, and treating hospital and country were extracted from the registry. Hospitals participating in PROM collection routinely administered the Core Outcome Measures Index (COMI) and the EuroQol-5D (EQ-5D) as component of the registry, and some hospitals additionally sent the Oswestry Disability Index (ODI) on their own initiative. PROMs could be administered either electronically or on paper. Completion of PROMs was evaluated in terms of any PROM completion and further categorized into completion of baseline PROMs, follow-up PROMs, and paired PROMs, meaning that a patient completed both a baseline and a follow-up PROM. PROMs were provided to patients at baseline, and at 3 and 12 months follow-up. As this study aimed to evaluate participation in routine PROM collection after PROM implementation rather than the clinical outcomes assessed by PROMs, a patient was considered to have completed PROMs at a specific timepoint if at least one of the available PROM instruments (COMI, EQ-5D, or ODI) was completed.

Additionally, hospitals participating in the Spine Tango Registry, included in this study, were asked to fill out a questionnaire regarding local PROM collection strategies at their hospital, including administration methods, estimated use, perceived patient compliance and feasibility of each method, reminder systems, staff involvement, and other strategies used to increase patient compliance with PROMs (Appendix A).

### 2.4 Statistical analysis

Descriptive statistics were used to summarize baseline characteristics. The completion of any PROMs in general and separately the completion of baseline PROMs, follow-up PROMs, and paired PROMs were reported using numbers and percentages and compared across diagnostic groups using chi-squared tests. Additionally, mixed-effects logistic regression models were applied to assess the association between patient characteristics and PROM completion. Fixed effects were age, diagnosis group (LRS, LSS or both), and whether fusion surgery was performed, and were reported as odds ratios (ORs) with 95% confidence intervals (95% CIs). Random intercepts were included for hospital and country of treatment and were reported using standard deviations (SDs). The contribution of random effects to the model fit was assessed using likelihood ratio tests (LRTs). In addition, the proportion of variation in PROM completion attributable to differences between hospitals was quantified using the intraclass correlation coefficient (ICC), which was derived from the variance of the hospital random intercept. If a patient underwent multiple procedures, double inclusion of a patient in the models was prevented by selecting one procedure using the following logic: 1) earliest surgery with paired PROM completion; 2) earliest surgery with any PROM completion; 3) earliest surgery. Furthermore, a sub-analysis was performed among patients who were sent ePROMs, thereby excluding patients from hospitals that had implemented ePROMs but to whom no PROMs or only paper PROMs were sent. P-values ≤0.05 were considered statistically significant.

## 3 Results

### 3.1 Demographics

A total of 1810 patients who underwent 1827 procedures were included, with a small number of patients receiving multiple procedures. Procedures were performed across 23 hospitals located in 5 countries, predominantly in Switzerland (76.5%) and the United Kingdom (17.5%). The mean age across all procedures was 64.2 years and 51.8% of patients were female. Most procedures were performed for LSS alone (61.2%) and decompression without fusion was the most common approach (63.0%) (Table 1).

**Table 1:** Baseline characteristics of included procedures.

	LRS (n = 450)	LSS (n = 1119)	LRS & LSS (n = 258)	Total (n = 1827)
Mean age, years	53.0	69.1	62.2	64.2
Female, n (%)	225 (50.0%)	588 (52.5%)	134 (51.9%)	947 (51.8%)
Country, n (%)				
Ireland				55 (3.0%)
Italy				2 (0.1%)
Portugal				52 (2.8%)
Switzerland				1398 (76.5%)
United Kingdom				320 (17.5%)
Number of segments affected, n (%)				
1	300 (67.0%)	531 (47.6%)	135 (53.4%)	966 (53.2%)
2-3	144 (32.1%)	535 (48.0%)	106 (41.9%)	785 (43.2%)
≥ 4	4 (0.9%)	49 (4.4%)	12 (4.7%)	65 (3.6%)
Unknown	2 (0.4%)	4 (0.4%)	5 (1.9%)	11 (0.6%)
Received fusion n (%)				
No	332 (76.7%)	540 (55.0%)	167 (70.8%)	1039 (63.0%)
Yes	101 (23.3%)	441 (45.0%)	69 (29.2%)	611 (37.0%)
Unknown	17 (3.8%)	138 (12.3%)	22 (8.5%)	177 (9.7%)
Received previous surgery at any level, n (%)	143 (31.8%)	320 (28.6%)	66 (25.6%)	529 (29.0%)

LRS = lumbosacral radicular syndrome, LSS = lumbar spinal stenosis.

### 3.2 PROM completion rates

Overall, 67.3% of patients completed at least one PROM, with 57.9% of patients completing a baseline PROM and 53.3% of patients completing a follow-up PROM. Paired baseline and (any) follow-up PROMs were available for 44.4% of patients. PROM completion rates declined with increasing follow-up duration. In univariate analyses, completion rates differed significantly across diagnostic groups (LRS, LSS and LRS & LSS) at almost all time points (p < 0.001), except for >6 months follow-up, where the difference was not statistically significant (p = 0.058). Patients undergoing surgery for LSS consistently had the lowest completion rates (Table 2). Furthermore, completion rates varied across countries, with country-specific completion rates presented in Table 3.

**Table 2:** PROM completion across timepoints and diagnoses.

	Total (n = 1810)	Diagnostic groups			
		LRS (n = 445)	LSS (n = 1110)	LRS & LSS (n = 255)	p-value^‡^
Any PROM	1219 (67.3%)	367 (82.5%)	645 (58.1%)	207 (81.2%)	<0.001*
Baseline PROM	1048 (57.9%)	319 (71.7%)	554 (49.9%)	175 (68.6%)	<0.001*
Follow-up PROM	964 (53.3%)	293 (65.8%)	493 (44.4%)	178 (69.8%)	<0.001*
3 months**^†^**	574 (31.7%)	202 (45.4%)	249 (22.4%)	123 (48.2%)	<0.001*
>6 months**^†^**	433 (25.1%)	117 (27.8%)	246 (23.2%)	70 (29.0%)	0.058
Paired PROM	804 (44.4%)	250 (56.2%)	405 (36.5%)	149 (58.4%)	<0.001*

**^†^‘**3 months’ refers to PROMs completed at the scheduled 3-month follow-up timepoint, ‘>6 months’ refers to PROMs completed between 6 and 12 months postoperatively. Only patients with sufficient time between procedure date and data extraction were included in the >6 months group, resulting in the following group sizes: LRS 421 patients, LSS 1061 patients, LRS & LSS 241 patients. **^‡^**Chi-square tests were used to compare diagnostic groups, *indicates p < 0.05.

PROM = patient-reported outcome measure, LRS = lumbosacral radicular syndrome, LSS = lumbar spinal stenosis.

**Table 3:** Completion rates by timepoint and country.

	Ireland	Portugal	Switzerland	United Kingdom	Italy
Any PROM	51 (92.7%)	41 (80.4%)	850 (61.5%)	265 (83.1%)	1 (100%)
Baseline PROM	50 (90.9%)	39 (76.5%)	805 (58.2%)	154 (48.3%)	N/A
Follow-up PROM	24 (43.6%)	23 (45.1%)	693 (50.1%)	223 (70.0%)	1 (100%)
Paired PROM	23 (41.8%)	21 (41.2%)	648 (46.9%)	112 (35.1%)	N/A

N/A: No baseline PROMs were sent. PROM = patient-reported outcome measure.

### 3.3 Predictors of PROM completion

In the adjusted analyses, age was significantly associated with PROM completion. Compared to the oldest group of 80 years and over, all age groups except the youngest group of 0 to 39 years old had a significantly higher odds of ‘completing any PROM’ (Table 4). The odds of ‘completing any PROM’ compared to the group of 80 years and older increased progressively across age groups, reaching statistical significance from the 40-49 year group and peaking in patients aged 60-69 years (OR 2.329, p < 0.001), before declining in the 70-79 year group, while remaining significantly higher compared to patients aged 80 years and older. A similar pattern was observed for ‘completion of baseline PROMs’. This trend was absent considering associations between age group and ‘completion of follow-up PROMs’ or ‘completion of paired baseline and follow-up PROMs’ (Table 4).

**Table 4:** Results of mixed-effects logistic regression models.

	Variables	Estimate	OR (95% CI)	p-value	SD
**Any PROM**	*Fixed effects*				
	Age (ref. ≥80 y)				
	0-39	0.064	1.066 (0.56-2.04)	0.848	
	40-49	0.660	1.935 (1.07-3.48)	0.028*	
	50-59	0.811	2.251 (1.41-3.59)	0.001*	
	60-69	0.845	2.329 (1.51-3.58)	<0.001*	
	70-79	0.594	1.811 (1.21-2.71)	0.004*	
	Diagnosis (ref. LRS)				
	LSS	−0.387	0.679 (0.46-1.00)	0.047*	
	LRS & LSS	−0.475	0.622 (0.38-1.01)	0.054	
	Fusion surgery (ref. no)				
	Yes	−0.419	0.658 (0.42-1.02)	0.060	
	Unknown	−0.463	0.629 (0.36-1.20)	0.103	
	*Random effects*				
	Hospital				2.339
	Country				0.001

**Baseline PROM**	*Fixed effects*				
	Age (ref. ≥80 y)				
	0-39	0.463	1.589 (0.89-2.83)	0.116	
	40-49	0.692	1.997 (1.18-3.38)	0.010*	
	50-59	0.711	2.037 (1.32-3.14)	0.001*	
	60-69	0.813	2.254 (1.51-3.37)	<0.001*	
	70-79	0.637	1.891 (1.30-2.76)	0.001*	
	Diagnosis (ref. LRS)				
	LSS	−0.126	0.881 (0.63-1.23)	0.457	
	LRS & LSS	−0.582	0.559 (0.37-0.85)	0.007*	
	Fusion surgery (ref. no)				
	Yes	−0.386	0.680 (0.47-0.99)	0.044*	
	Unknown	−0.521	0.594 (0.35-1.00)	0.051	
	*Random effects*				
	Hospital				2.193
	Country				0.000

**Follow-up PROM**	*Fixed effects*				
	Age (ref. ≥80 y)				
	0-39	−0.277	0.758 (0.44-1.29)	0.309	
	40-49	0.428	1.535 (0.91-2.58)	0.107	
	50-59	0.226	1.254 (0.81-2.01)	0.304	
	60-69	0.268	1.308 (0.88-1.95)	0.187	
	70-79	0.272	1.312 (0.90-1.92)	0.163	
	Diagnosis (ref. LRS)				
	LSS	−0.272	0.762 (0.55-1.05)	0.100	
	LRS & LSS	0.025	1.025 (0.69-1.53)	0.903	
	Fusion surgery (ref. no)				
	Yes	−0.304	0.738 (0.53-1.03)	0.073	
	Unknown	−0.083	0.920 (0.53-1.61)	0.770	
	*Random effects*				
	Hospital				1.924
	Country				0.000

**Paired PROM**	*Fixed effects*				
	Age (ref. ≥80 y)				
	0-39	−0.015	0.985 (0.58-1.67)	0.955	
	40-49	0.487	1.628 (0.99-2.68)	0.056	
	50-59	0.216	1.241 (0.81-1.89)	0.315	
	60-69	0.305	1.357 (0.92-2.01)	0.126	
	70-79	0.374	1.453 (1.00-2.11)	0.051	
	Diagnosis (ref. LRS)				
	LSS	−0.107	0.899 (0.66-1.23)	0.503	
	LRS & LSS	−0.206	0.814 (0.56-1.19)	0.289	
	Fusion				
	Yes	−0.274	0.760 (0.55-1.04)	0.090	
	Unknown	−0.234	0.791 (0.45-1.38)	0.412	
	*Random effects*				
	Hospital				1.885
	Country				0.004

* indicates p < 0.05. PROM = patient-reported outcome measure, LRS = lumbosacral radicular syndrome, LSS = lumbar spinal stenosis.

Regarding diagnosis, after adjustment for age and other variables in the model, patients with LSS were significantly less likely to ‘complete any PROM’ compared to patients with LRS (OR = 0.679, p = 0.047) and patients with combined LRS and LSS had significantly lower odds of ‘completing baseline PROMs’ compared to patients with LRS alone (OR 0.559, p = 0.007). No other significant associations between diagnosis and PROM completion were found (Table 4).

Fusion surgery was associated with significantly lower odds of ‘completing baseline PROMs’ compared to decompression without fusion surgery (OR 0.680, p = 0.044), although this association was not significant for the completion of any PROMs in general, follow-up PROMs or paired PROMs (Table 4).

**Fig. 1:**
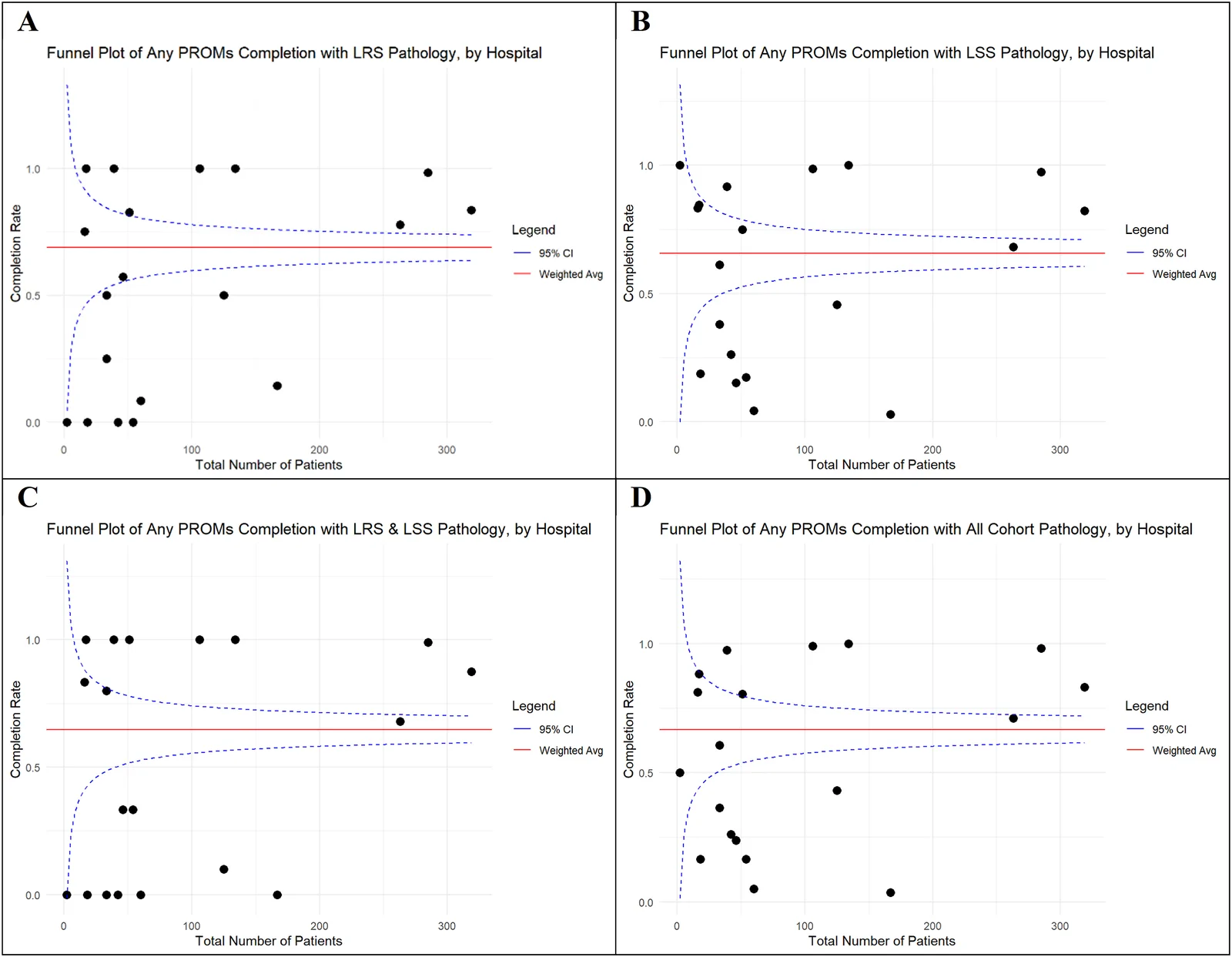
Funnel plots of the completion rate of any PROMs (baseline or follow-up) by hospital in **(A)** LRS patients, **(B)** LSS patients, **(C)** patients with both LRS and LSS, **(D)** all patients. The dashed blue lines indicate the 95% confidence interval limits, the solid red line represents the weighted average. Abbreviations: PROM = patient-reported outcome measure, LRS = lumbosacral radicular syndrome, LSS = lumbar spinal stenosis.

Considerable variability in PROM completion was observed between hospitals, with the standard deviations (SD) of hospital random effect ranging from 1.885 for paired PROMs to 2.339 for any PROMs. As these values are expressed on the log-odds scale, this corresponds to approximately a seven-to tenfold difference in odds of PROM completion between hospitals differing one SD. Furthermore, the intraclass correlation coefficient (ICC) for hospital random effect in the model for ‘any PROM completion’ was 0.62, indicating that 62% of the total variation in PROM completion is attributable to differences between hospitals. In contrast, country-level variation was minimal, with SDs ranging from 0.000 for baseline and follow-up PROMs to 0.004 for paired PROMs (Table 4). A likelihood ratio test demonstrated that inclusion of the random effects significantly improved the fit of all models (p < 0.001). The rates of ‘any PROM completion’ by hospital were visualized using funnel plots (Fig. 1).

The aforementioned analyses encompass all results collected in the hospitals from the timepoint at which they reported initiating the distribution of PROMs. It is conceivable that this may present a biased representation, as PROMs may not have been sent to all patients, or PROMs may have been distributed via conventional mail and subsequently not recorded in the database. Therefore, a sub-analysis including only patients who were sent at least one electronic PROM was conducted. The total number of patients in this sub-analysis is smaller, namely 687 (Appendix B). The lower completion rate of PROMs in the LSS diagnosis group was not observed in this sub-analysis. The trends in odds ratios concerning the predicting factors were comparable to the analysis in the whole group, although the results yielded fewer statistically significant associations.

### 3.4 PROM collection strategies

A total of 14 out of 23 hospitals responded to the survey on PROM collection strategies. Of these, 3 did not answer any questions except hospital name and country, and 1 hospital responded not to be familiar with PROMs, and these were therefore excluded from the analysis. Consequently, a total of 10 hospitals, all located in Switzerland, were used for the exploratory analysis on PROM strategies.

The most widely adopted PROM administration methods were electronic by email (90%) and/or text message (80%) (Table 5). Likewise, among hospitals using these methods, electronic via email (75.0% of use) and/or text message (72.5% of use) were also the most intensively used approaches.

Physicians from the participating hospitals who received the survey were asked to rate the perceived patient compliance and feasibility of each PROM administration method on a scale from 0 to 10 points. According to the physicians, all administration methods had a median rating of at least 7 points in terms of patient compliance. The electronic options (text message, email or tablet/desktop in the hospital) were rated by the physicians as good in terms of patient compliance (7/10 points), whereas filling in PROMs on paper administered to the patients in the hospital was perceived to be best in terms of patient compliance (9/10 points). In contrast, physicians considered electronic text message to be the most feasible administration method (7.5 points), followed by both on paper options (on paper in the hospital and on paper by letter, 7 points). Administering PROMs electronically using a tablet or desktop in the hospital was considered least feasible (4.5 points) (Table 5).

**Table 5:** Hospitals’ use and views on different PROM administration methods.

	Hospitals using method, n (%)	% of use among users, median (IQR) (n = 2-9)	View on compliance, median (IQR) (n = 5-7)	View on feasibility, median (IQR) (n = 5-7)
Electronic: text message with the link to PROMs	8 (80%)	72.5% (40-85%)	7 (5-8)	7.5 (5-10)
Electronic: email with the link to PROMs	9 (90%)	75.0% (45-95%)	7 (5-8)	6 (5-10)
Electronic: tablet or desktop in the hospital	2 (20%)	30.0% (10-50%)	7 (0-8)	4.5 (2-5)
On paper in the hospital	6 (60%)	12.5% (5-80%)	9 (8-9.5)	7 (6-10)
On paper by letter	2 (20%)	52.5% (15-90%)	8 (3.5-9)	7 (3.5-9)
By phone interview	4 (40%)	10.0% (10-15%)	8 (7-9)	6 (4-8)

Three hospitals reported reminding patients beyond automatic electronic reminders, for example using phone calls. Additionally, three hospitals had dedicated staff to support patients with PROMs and improve compliance, specifically a clinical research coordinator or a secretary.

In the open-ended questions asking about strategies used to encourage patients to fill in PROMs and increase completion rates, hospitals emphasized the importance of adequately informing patients about PROMs during the patient's visit, including an explanation of their purpose, and handing out the required PROMs to patients during their visit. Additionally, some hospitals reported reminding patients about PROMs using automated email reminders or phone calls. Flexibility in PROM administration methods was also emphasized, for example by offering patients multiple options to complete PROMs, sending PROMs by post if electronic PROMs are not completed, and in elderly patients using paper PROMs or phone calls rather than electronic PROMs.

## 4 Discussion

This study evaluated PROM completion rates and associated patient- and hospital-level factors in a multicenter lumbar spine surgery cohort. The data presented in this study demonstrated that approximately two-thirds of patients completed at least one PROM, whereas less than half had both a baseline and a follow-up PROM available. Moreover, PROM completion decreased considerably with increasing follow-up duration. While patient characteristics, particularly age, showed significant associations with PROM completion, there was also substantial variability between hospitals. Sixty-two percent of the variation in PROMs completion was demonstrated to be due to the specific hospital at which the patient received the treatment, indicating an important role for hospital-specific determinants.

The overall PROM completion rates observed in this study are broadly in line with those reported in previous spine studies, although substantial variability exists across studies. Johnson et al. demonstrated that between 2020 and 2022, 68.4% of all newly presenting spine patients at their clinic completed PROMs at their first visit (Johnson et al., 2024). Likewise, unpublished data from Van Grafhorst et al. at a single Dutch center showed that baseline PROM completion among lumbar surgical spine patients with access to digital PROM collection increased from 6.8% in 2019 to 60.5% in 2023, thereby being comparable to the baseline completion rates in our cohort. However, in this cohort, 45.3% of patients completed 2-month follow-up PROMs, which declined to only 0.8% at 6 months (Unpublished data). Ifearulundu et al. demonstrated considerably higher completion rates of 84.5% at baseline, decreasing to 54.4% at 3 months and 45.6% at 12 months (Ifearulundu et al., 2022). In contrast to these institutional studies, Scandinavian national spine registries have reported substantially higher completion rates during follow-up (Lønne et al., 2019), with Swespine achieving 73% completion at 1 year follow-up and 65% at two years follow-up (Swespine). Together, these findings demonstrate that although PROM completion generally declines with increasing follow-up duration, maintaining substantial follow-up is feasible in some healthcare settings.

The decline in PROM completion with increasing follow-up duration is particularly relevant because meaningful evaluation of surgical outcomes requires assessment beyond the immediate postoperative period. Traditionally, a minimum follow-up duration of two years has been considered necessary for PROM assessment after spine surgery to adequately capture postoperative recovery, although more recent evidence suggests that one year PROMs might also be sufficient (Staartjes et al., 2019; Parai et al., 2019). Nevertheless, in our study, only 25% of patients completed a PROM beyond six months postoperatively, indicating that this clinically relevant follow-up duration is not sufficiently achieved.

Adequate PROM completion is essential to ensure valid and representative outcome assessment (Zini and Banfi, 2021). The observed associations between PROM completion and both patient and hospital characteristics suggest that patients with available PROM data may not fully represent the overall surgical population, indicating a potential risk of non-response bias in outcome analyses. Moreover, previous studies have suggested that patients who are satisfied with their treatment and outcomes are more likely to complete PROMs compared to those who are dissatisfied, thereby resulting in an overrepresentation of patients with favorable outcomes (Levens et al., 2023). These findings emphasize the importance of optimizing PROM collection to ensure reliable interpretation of outcomes. One potential strategy may be for hospitals to actively support PROM collection in a clearly defined and representative sub-cohort rather than collecting limited data from all patients, as more intensive follow-up might improve data completeness and thereby reduce bias.

Our study found that age was significantly associated with PROM completion, with completion rates increasing progressively from younger to middle-aged patients, peaking in patients aged 60-69 years, and declining thereafter in older patients. This age-related pattern has also been observed in previous studies. A systematic review in orthopedic surgery patients identified both younger and older age as risk factors for lower PROM completion (Levens et al., 2023). Similarly, other studies have reported that PROM completion rates were highest in the middle-aged subgroup, with lowest completion rates in the youngest and oldest subgroups, although the peak age varied from 52 years to 75 years old (Vilkki et al., 2025; Nikkhah et al., 2025). These findings suggest that age-specific barriers might affect PROM completion, thereby highlighting the need for tailored interventions to improve PROM completion rates across different age groups.

Although the univariate analyses suggested significant differences in PROM completion between diagnostic groups, these associations were less pronounced after adjustment for other patient- and hospital-level factors. This suggests that the differences between diagnostic groups may partly reflect differences in patient characteristics and hospital factors across diagnostic groups rather than diagnosis itself. Nevertheless, after adjustment, patients with LSS still remained less likely to complete any PROMs and patients with combined LRS and LSS had lower odds of completing baseline PROMs compared to patients with LRS alone. One possible explanation might relate to the difference in symptom progression between LRS and LSS. Specifically, patients with LRS typically experience a more acute onset of symptoms, whereas LSS is characterized by a chronic, gradually progressive course (Strayer, 2005). Evidence from previous studies suggests that patients might perceive less value in completing PROMs if their symptoms have not changed much or if symptoms are recurrent (Unni et al., 2023; Meerhoff et al., 2021; Hamitouche et al., 2025). Moreover, studies have shown that patients with LRS are generally more satisfied with their treatment (Joelson et al., 2023; Macki et al., 2019), which has also been previously associated with higher PROM completion rates in other orthopedic populations (Lindman et al., 2020; Kim et al., 2004).

Furthermore, our study demonstrated that fusion surgery was associated with worse baseline PROM completion, whereas no significant associations were observed for follow-up PROM completion. This finding contradicted our initial expectations, as we hypothesized that the typically more extensive follow-up schedules after fusion surgery would increase patients’ long-term engagement with healthcare and thereby increase follow-up PROM completion. Nevertheless, this finding may reflect similar mechanisms to those described for diagnostic groups, since fusion surgery in patients with LRS or LSS is often performed in the setting of recurrent disease or previous decompression or discectomy without fusion (Lei et al., 2023; Sharif et al., 2020). Previous surgery has been associated with lower PROM completion in subsequent procedures (Schamber et al., 2013), although these findings are inconsistent across studies, with another study reporting higher completion rates at 12 months follow-up after previous surgery (Ifearulundu et al., 2022).

Our study demonstrated substantial variability in PROM completion rates between hospitals, even after adjustment for patient characteristics. The large standard deviation of hospital random effect and the high ICC demonstrate that, while individual patient characteristics certainly play a role, the majority of the variability in PROM completion was attributable to hospital-level factors. This indicates that organizational factors and processes, but also commitment and motivation from hospitals and physicians, play a crucial role in determining PROM completion. Importantly, this also suggests that with appropriate interventions, achieving higher PROM completion rates is in practice feasible, as some hospitals did achieve very high completion rates.

The exploratory survey conducted among participating hospitals further supports this interpretation, demonstrating considerable variation in PROM strategies between hospitals. Electronic PROM administration via email and text messaging were the most widely adopted and most intensively used approaches, whereas paper-based methods, phone interviews and in-hospital devices were less commonly and less intensively used. Nevertheless, the large IQRs indicate considerable variation between hospitals. Despite their lower use, paper PROMs completed at the hospital or sent via post were perceived to result in the highest patient compliance, and were together with electronic text messaging also considered to be the most feasible approaches. This discrepancy between actual use and perceived effectiveness suggests that, although PROM collection is increasingly digitalized and ePROMs offer advantages with regard to costs and administrative burden (Meirte et al., 2020), paper-based methods remain an important complementary option. Open-ended responses further emphasized the importance of flexibility, indicating that offering alternative administration methods may help to engage patients who do not respond to ePROMs and thereby improve the overall PROM completion rates. Moreover, three hospitals reported having dedicated staff to support PROM administration. Previous research by Marjamaa et al. has suggested that dedicated staff may facilitate PROM completion, as their study reported an overall completion rate of 58% with rates reaching up to 90% in hospitals with dedicated staff (Marjamaa et al., 2023). Another presumable key element in ensuring a high response rate is monitoring response rates overall, as well as specifically monitoring whether patients respond or not, and taking action if they do not.

In contrast to the substantial variability between hospitals, the estimated variability at the country level was negligible in our models. However, this finding should be interpreted with caution, as the number of countries in our study was very small. This limits the ability of mixed-effects models to detect between-country variability and may result in downward bias of the variance estimates, even when true variability exists (McNeish and Stapleton, 2016). Notably, most hospitals and patients included in this study were from Switzerland, although the Spine Tango Registry includes hospitals from 20 different countries. This predominance of Swiss hospitals likely reflects a combination of their strong national emphasis on quality monitoring, which is mandatory in Switzerland (Swiss Confederation), as well as the Swiss origin of the Spine Tango registry (Melloh et al., 2008), which might have resulted in broader adoption among Swiss hospitals. Nevertheless, low PROM completion rates observed across many participating hospitals indicate that the availability of a PROM collection system alone is insufficient to ensure successful PROM implementation. In addition to hospital-level initiatives, broader healthcare system support may therefore be important to facilitate routine PROM collection. For example, in England, PROM collection for primary hip and knee arthroplasty is part of a national quality program in which the reimbursement amount is linked to predefined quality criteria, including achieving a minimum PROM completion rate of 50% (Matthews and Evans, 2023). A recent study reported that procedures within this program were associated with more frequent and standardized PROM collection (Matthews and Evans, 2023). This illustrates how healthcare authorities may facilitate the routine implementation of PROM collection through policy and reimbursement initiatives.

This study has several important strengths. First of all, this study was based on data from a large, international spine registry used in routine clinical practice, thereby enhancing the external validity and clinical relevance of findings. Secondly, to our knowledge, this is the first multicenter study to systematically evaluate PROM completion rates and associated patient- and hospital-level factors in spine surgery patients. The inclusion of multiple centers enabled direct comparison between hospitals, which demonstrated the large variability in PROM completion across hospitals. Third, the addition of an exploratory hospital survey provided insight into hospital-specific PROM collection strategies, thereby offering context information for the registry-based findings. Some limitations should also be considered. The majority of patients and participating hospitals were from Switzerland, which may limit the generalizability of findings to other healthcare systems and restricted assessment of between-country variability. In addition, the assessment of patient-level determinants was limited to the variables routinely collected within the Spine Tango registry. Several potentially relevant patient characteristics, such as comorbidities, educational level, and socioeconomic status were not collected and could therefore not be evaluated. Lastly, although the exploratory hospital survey provided valuable insight into PROM collection strategies at hospitals, these data were not linked to hospital-level PROM completion rates. Consequently, although we demonstrated clear variability in PROM completion rates and PROM collection strategies between hospitals, the relationship between specific collection strategies and PROM completion could not be assessed. Future studies should focus on identifying which hospital strategies are most effective in improving PROM completion and on identifying a broader range of patient characteristics associated with PROM completion. Such insights may help inform targeted interventions to optimize PROM collection and thereby enhance the completeness and validity of outcome assessments in routine clinical practice.

## 5 Conclusion

PROM completion in routine lumbar spine surgery care remains incomplete, particularly for long-term follow-up and paired PROM assessments. While patient characteristics such as age and surgical factors were associated with completion, the substantial variability between hospitals highlights the crucial role of hospitals in improving PROM completion. Therefore, improving PROM completeness in spine surgery requires primarily hospital-level interventions to ensure reliable and representative outcome measurement.

## Authors’ contributions

All authors contributed to the conception and design of the study. CLAVL and JM interpreted the results. JM wrote the first draft of the manuscript, which was critically reviewed and edited by CLAVL, EA, JMPG, and CH. CLAVL supervised the study. All authors read and approved the final manuscript.

## Funding sources

This research did not receive any specific grant from funding agencies in the public, commercial, or not-for-profit sectors.

## Declaration of competing interests

The authors declare the following financial interests/personal relationships which may be considered as potential competing interests: CLAVL reports a relationship with Cervical Spine Research Society that includes: board membership and funding grants. CLAVL reports a relationship with Netherlands Neurosurgical Society (NVvN) that includes: board membership. CLAVL reports a relationship with EUROSPINE The Spine Society of Europe that includes: board membership and funding grants. CLAVL reports a relationship with Rijndam Rehabilitation that includes: consulting or advisory. CLAVL reports a relationship with Covidien that includes: funding grants. CLAVL reports a relationship with YM Fund that includes: funding grants. CLAVL reports a relationship with Achmea Health Insurance that includes: funding grants. CH reports a relationship with EUROSPINE The Spine Society of Europe that includes: employment. EA reports a relationship with EUROSPINE The Spine Society of Europe that includes: employment. If there are other authors, they declare that they have no known competing financial interests or personal relationships that could have appeared to influence the work reported in this paper.
