# Supplementary material for: Patient-reported outcome measure completion in lumbar spine surgery: Rates and determinants in a multicenter registry
Source: Brain Spine. 2026 Aug 18;6:106286. doi: 10.1016/j.bas.2026.106286 (PMC13524754; doi:10.1016/j.bas.2026.106286)
Supplement: Supplementary file 1 [file mmc1.docx]

**Appendix A – Hospital survey on PROM collection strategies**

Hospital name

Hospital country

1. Does your hospital collect PROMs?
   - Yes
   - No
   - Will start soon

**If the answer to Question 1 was “No”:**

2. What are the reasons for not collecting PROMs? (multiple answers possible)
   - High additional efforts
   - No interest
   - Other / to specify

**If the answer to Question 1 was “Yes” or “Will start soon”:**

3. Does/will your staff members inform patients about PROMs to get their consent and assure compliance?
   - Yes
   - No
4. Which PROMs administration methods does/will your hospital use and can you estimate the level of patient compliance and hospital’s feasibility in terms of the effort (give a value between 0 and 10, with 0 being not compliant/feasible and 10 being most convenient for the patient/absolutely feasible)

| **Method** | **Used/will be used (indicate % of use)** | **Not used (tick box if applicable)** | **Your view on patient compliance (0-10 points)** | **Your view on feasibility**  **(0-10 points)** |
| --- | --- | --- | --- | --- |
| Electronic: text message |  |  |  |  |
| Electronic: email |  |  |  |  |
| On paper in the hospital |  |  |  |  |
| On paper by letter |  |  |  |  |
| By phone interview |  |  |  |  |
| Other / to specify |  |  |  |  |

5. Which strategies do you use to encourage patients to fill in the forms, including those at follow-ups?
6. Do you remind patients beyond the electronic automatically sent reminders?
7. Is there any dedicated staff involved in supporting patients with PROMs, including follow-up?
8. Are there any specific strategies that you find effective in achieving higher completion rates in PROMs?
