# Supplementary material for: Patient-reported outcome measure completion in lumbar spine surgery: Rates and determinants in a multicenter registry
Source: Brain Spine. 2026 Aug 18;6:106286. doi: 10.1016/j.bas.2026.106286 (PMC13524754; doi:10.1016/j.bas.2026.106286)
Supplement: Supplementary file 2 [file mmc2.docx]

**Appendix B – Results of subgroup analysis**

**Table B.1**: ePROM completion rates per timepoint and diagnosis

|  | **Total**  **(n = 687)** | **Diagnostic groups** | | | |
| --- | --- | --- | --- | --- | --- |
|  |  | **LRS**  **(n = 188)** | **LSS**  **(n = 420)** | **LRS & LSS**  **(n = 79)** | **p-value^‡^** |
| Any ePROM | 576 (83.8%) | 161 (85.6%) | 348 (82.9%) | 67 (84.8%) | 0.669 |
| Baseline ePROM | 446 (64.9%) | 126 (67.0%) | 274 (65.2%) | 46 (58.2%) | 0.380 |
| Follow-up ePROM | 471 (68.6%) | 131 (69.7%) | 286 (68.1%) | 54 (68.4%) | 0.926 |
| 3 months**^†^** | 165 (24.0%) | 54 (28.7%) | 97 (23.1%) | 14 (17.7%) | 0.123 |
| >6 months**^†^** | 284 (42.1%) | 78 (41.9%) | 168 (40.9%) | 38 (49.4%) | 0.384 |
| Paired ePROM | 342 (49.8%) | 97 (51.6%) | 212 (50.5%) | 33 (41.8%) | 0.308 |

**^†^‘**3 months’ refers to PROMs completed at the scheduled 3-month follow-up timepoint, ‘ >6 months’ refers to PROMs completed between 6 and 12 months postoperatively. Only patients with sufficient time between procedure date and data extraction were included in the >6 months group, resulting in the following group sizes: LRS 186 patients, LSS 411 patients, LRS & LSS 77 patients. **^‡^**Chi-square tests were used to compare diagnostic groups.

PROM = patient-reported outcome measure, LRS = lumbosacral radicular syndrome, LSS = lumbar spinal stenosis.

**Table B.2**: results of mixed effects logistic regression models

|  | **Predictor** | **Estimate** | **OR (95% CI)** | **p-value** | **SD** |
| --- | --- | --- | --- | --- | --- |
| **Any PROM** | *Fixed effects* |  |  |  |  |
|  | Age (ref. ≥80) |  |  |  |  |
|  | 0-39 | -0.263 | 0.769 (0.32-1.86) | 0.561 |  |
|  | 40-49 | 0.403 | 1.497 (0.62-3.59) | 0.366 |  |
|  | 50-59 | 0.624 | 1.867 (0.88-3.94) | 0.102 |  |
|  | 60-69 | 0.365 | 1.441 (0.73-2.83) | 0.288 |  |
|  | 70-79 | 0.524 | 1.689 (0.87-1.19) | 0.123 |  |
|  | Diagnosis (ref. LRS) |  |  |  |  |
|  | LSS | -0.239 | 0.787 (0.45-1.36) | 0.392 |  |
|  | LRS & LSS | -0.244 | 0.783 (0.40-1.54) | 0.479 |  |
|  | Fusion surgery (ref. no) |  |  |  |  |
|  | Yes | -0.264 | 0.768 (0.42-1.39) | 0.381 |  |
|  | Unknown | -0.127 | 0.881 (0.35-2.22) | 0.788 |  |
|  | *Random effects* |  |  |  |  |
|  | Hospital |  |  |  | 0.515 |
|  | Country |  |  |  | 0.001 |
| **Baseline** | *Fixed effects* |  |  |  |  |
| **PROM** | Age (ref. ≥80) |  |  |  |  |
|  | 0-39 | 0.488 | 1.629 (0.74-3.59) | 0.226 |  |
|  | 40-49 | 0.728 | 2.070 (0.99-4.35) | 0.055 |  |
|  | 50-59 | 0.551 | 1.734 (0.92-3.28) | 0.090 |  |
|  | 60-69 | 0.611 | 1.842 (1.01-3.38) | 0.048* |  |
|  | 70-79 | 0.766 | 2.152 (1.19-3.90) | 0.012* |  |
|  | Diagnosis (ref. LRS) |  |  |  |  |
|  | LSS | 0.100 | 1.105 (0.71-1.72) | 0.659 |  |
|  | LRS & LSS | -0.659 | 0.518 (0.29-0.91) | 0.022* |  |
|  | Fusion surgery (ref. no) |  |  |  |  |
|  | Yes | 0.030 | 1.031 (0.64-1.67) | 0.903 |  |
|  | No | 0.094 | 1.099 (0.48-2.52) | 0.824 |  |
|  | *Random effects* |  |  |  |  |
|  | Hospital |  |  |  | 0.876 |
|  | Country |  |  |  | 0.000 |
| **Follow-up** | *Fixed effects* |  |  |  |  |
| **PROM** | Age (ref. ≥80) |  |  |  |  |
|  | 0-39 | -0.890 | 0.411 (0.19-0.90) | 0.026* |  |
|  | 40-49 | -0.198 | 0.820 (0.39-1.72) | 0.599 |  |
|  | 50-59 | -0.163 | 0.850 (0.45-1.60) | 0.615 |  |
|  | 60-69 | -0.192 | 0.825 (0.45-1.51) | 0.532 |  |
|  | 70-79 | 0.048 | 1.049 (0.58-1.89) | 0.872 |  |
|  | Diagnosis (ref. LRS) |  |  |  |  |
|  | LSS | -0.445 | 0.641 (0.41-1.00) | 0.052 |  |
|  | LRS & LSS | -0.230 | 0.795 (0.45-1.40) | 0.427 |  |
|  | Fusion surgery (ref. no) |  |  |  |  |
|  | Yes | -0.146 | 0.864 (0.53-1.42) | 0.562 |  |
|  | Unknown | -0.162 | 0.851 (0.39-1.84) | 0.682 |  |
|  | *Random effects* |  |  |  |  |
|  | Hospital |  |  |  | 0.670 |
|  | Country |  |  |  | 0.633 |
| **Paired** | *Fixed effects* |  |  |  |  |
| **PROM** | Age (ref. ≥80) |  |  |  |  |
|  | 0-39 | -0.297 | 0.743 (0.35-1.59) | 0.444 |  |
|  | 40-49 | 0.167 | 1.182 (0.59-2.37) | 0.638 |  |
|  | 50-59 | -0.080 | 0.923 (0.50-1.69) | 0.797 |  |
|  | 60-69 | 0.107 | 1.113 (0.63-1.98) | 0.716 |  |
|  | 70-79 | 0.344 | 1.410 (0.81-2.47) | 0.229 |  |
|  | Diagnosis (ref. LRS) |  |  |  |  |
|  | LSS | -0.172 | 0.842 (0.56-1.27) | 0.415 |  |
|  | LRS & LSS | -0.688 | 0.503 (0.29-0.88) | 0.016* |  |
|  | Fusion surgery (ref. no) |  |  |  |  |
|  | Yes | 0.042 | 1.043 (0.67-1.63) | 0.853 |  |
|  | Unknown | 0.010 | 1.010 (0.47-2.16) | 0.979 |  |
|  | *Random effects* |  |  |  |  |
|  | Hospital |  |  |  | 1.036 |
|  | Country |  |  |  | 0.002 |

* Indicates p <0.05. PROM = patient-reported outcome measure, LRS = lumbosacral radicular syndrome, LSS = lumbar spinal stenosis.
